# Integrative analysis of microRNAs and mRNAs reveals the regulatory networks of triterpenoid saponin metabolism in Soapberry (*Sapindus mukorossi* Gaertn.)

**DOI:** 10.3389/fpls.2022.1037784

**Published:** 2023-01-09

**Authors:** Yuanyuan Xu, Jiming Liu, Xiangqin Ji, Guochun Zhao, Tianyun Zhao, Xin Wang, Lixian Wang, Shilun Gao, Yingying Hao, Yuhan Gao, Yuan Gao, Xuehuang Weng, Liming Jia, Zhong Chen

**Affiliations:** ^1^ Key Laboratory of Silviculture and Conservation of the Ministry of Education, College of Forestry, Beijing Forestry University, Beijing, China; ^2^ National Energy R&D Center for Non-food Biomass, Beijing Forestry University, Beijing, China; ^3^ National Innovation Alliance of Sapindus Industry, Beijing Forestry University, Beijing, China; ^4^ Bioinformatics Analysis Department, Hangzhou KaiTai Biotechnology Co., Ltd, Hangzhou, Zhejiang, China; ^5^ Planning and Design Institute of Forest Products Industry, National Forestry and Grassland Administration, Beijing, China; ^6^ Research and Development Department, Yuanhua Forestry Biological Technology Co., Ltd., Sanming, Fujian, China; ^7^ Beijing Advanced Innovation Center for Tree Breeding by Molecular Design, Beijing Forestry University, Beijing, China

**Keywords:** *Sapindus mukorossi*, miRNA, triterpenoid saponin, biosynthesis, coexpression network

## Abstract

Triterpenoid saponin are important secondary metabolites and bioactive constituents of soapberry (*Sapindus mukorossi* Gaertn.) and are widely used in medicine and toiletry products. However, little is known about the roles of miRNAs in the regulation of triterpenoid saponin biosynthesis in soapberry. In this study, a total of 3036 miRNAs were identified, of which 1372 miRNAs were differentially expressed at different stages of pericarp development. Important KEGG pathways, such as terpenoid backbone biosynthesis, sesquiterpenoid and triterpenoid biosynthesis, and basal transcription factors were highlighted, as well the roles of some key miRNAs, such as ath-miR5021, han-miR3630-3p, and ppe-miR858, which may play important roles in regulating triterpenoid saponin biosynthesis. In addition, 58 miRNAs might participate in saponin biosynthesis pathways by predicting the targets of those miRNAs to 53 saponin biosynthesis structural genes. And 75 miRNAs were identified to potentially play vital role in saponin accumulation by targeting transcript factor genes, bHLH, bZIP, ERF, MYB, and WRKY, respectively, which are candidate regulatory genes in the pathway of saponin biosynthesis. The results of weighted gene coexpression network analysis (WGCNA) suggested that two saponin-specific miRNA modules and 10 hub miRNAs may participate in saponin biosynthesis. Furthermore, multiple miRNA–mRNA regulatory networks potentially involved in saponin biosynthesis were generated, e.g., ath-miR5021–*SmIDI2*/*SmGPS5*/*SmbAS1*/*SmCYP71D-3*/*SmUGT74G-2*, han-miR3630-3p–*SmCYP71A-14*/*SmbHLH54*/*SmMYB135*/*SmWRKY32*, and ppe-miR858–*SmMYB5*/*SmMYB32*. qRT-PCR analysis validated the expression patterns of nine miRNAs and 12 corresponding target genes. This study represents the first comprehensive analysis of miRNAs in soapberry and lays the foundation for further understanding of miRNA-based regulation in triterpenoid saponin biosynthesis.

## Introduction

Soapberry (*Sapindus mukorossi* Gaertn.) is an important economic agricultural product, which is widely used in biomedicine, bioenergy, and cosmetics (Zhao et al., 2019). The whole plant, including its fruit, root, bark, and leaves, has been used in traditional medicine in China (Xu et al., 2018). The crude protein content of soapberry is about 22%, which is comparable with protein-rich vegetables such as *Phaseolus legumin* and the pulses seeds meal proteins. And the essential amino acids in its globulin and albumin could meet the needs of infants aged 2–5 years recommended by FAO/WHO. These make the proteins from soapberry seeds potentially a source of protein nutrition (Yin et al., 2011). And the kernels of soapberry contain about 40% fatty acids and could be used to produce biodiesel and high-grade lubricants (Sun et al., 2017). In addition, previous studies indicated that soapberry accumulates up to 27% (w/w pericarp dry weight) triterpenoid saponins (Sun et al., 2017). Triterpenoid saponins consist of non-polar aglycones (triterpene) and one or more glycone (monosaccharide) moieties, which make them hydrophilic and hydrophobic. These properties make soapberry saponins excellent emulsifiers and foaming agents, with antibacterial, antitumor, hepatoprotective, antihyperglycemic, antidyslipidemic, and insecticidal activities (Upadhyay and Singh, 2012). More than 70 variant triterpenoid saponins have been isolated from soapberry and identified as oleanane-type, dammarane-type, tirucullane-type and lupane-type saponins, respectively (Xu et al., 2018). The triterpenoid saponin biosynthesis pathway is divided into three stages. In the early stage, isopentenyl diphosphate and its isomer dimethylallyl diphosphate are synthesized *via* the mevalonic acid (MVA) pathway in the cytosol and the 2-C-methyl-D-erythritol 4-phosphate (MEP) pathway in the plastid. In the intermediate stage, isopentenyl diphosphate and dimethylallyl diphosphate are catalyzed by the corresponding enzymes to produce 2,3-oxidesqualene. The late stage involves the synthesis of triterpenoid saponins from 2,3-oxidesqualene (Augustin et al., 2011; Zhao and Li, 2018; Xu et al., 2021a).

MicroRNAs (miRNAs) are a class of endogenous non-coding small RNAs (sRNAs), approximately 18–25 nucleotides in length, that perform vital regulatory roles in numerous biological and metabolic processes not only in animals, but also in plants (Song et al., 2019). In plants, miRNAs regulate target gene expression mainly *via* direct endonucleolytic cleavage or translational inhibition at the posttranscriptional level (Bartel, 2004). Studies have shown that miRNAs are involved in various biological processes in plants, including development, biotic and abiotic stress responses, pathogen defense, and the biosynthesis of secondary metabolites (Bologna and Voinnet, 2014; Ye et al., 2020). miRNAs can regulate the biosynthesis of triterpenoid saponin by directly targeting genes encoding biosynthetic enzymes, TFs, or other regulators. For example, miR854e in *Panax ginseng* was shown to target farnesyl pyrophosphate synthetase (*FPS*) in ginsenoside biosynthesis, while miR1439b and miR1439h were shown to target β-amyrin synthase (*β-AS*) (Mathiyalagan et al., 2013). Moreover, Wei et al. (2015) and Kajal and Singh (2017) identified several miRNAs involved in the triterpenoid saponin biosynthesis pathway in *Panax notoginseng* and *Chlorophytum borivilianum* by high-throughput sequencing. Although miRNAs related to triterpene biosynthesis have been identified, there have been no reports to date of miRNAs in soapberry.

In this study, to comprehensively investigate the regulatory mechanisms of triterpenoid saponin biosynthesis in soapberry, 24 sRNA libraries were constructed from soapberry pericarps at eight stages of development. We used bioinformatics tools to predict known and novel miRNAs and their potential targets. Further, the miRNA expression profiles were determined during the pericarp development process, and differential expression analysis and weighted gene coexpression network analysis (WGCNA) were performed to identify the miRNAs associated with saponin biosynthesis. Furthermore, miRNA coexpression networks and miRNA–mRNA interaction networks were constructed to characterize the key regulators in the saponin biosynthesis pathway. This study identified many miRNAs and their target genes, which not only provided the first comprehensive miRNA expression profile, but also built a foundation for future studies of miRNA-mediated regulation of saponin biosynthesis in soapberry.

## Materials and methods

### Plant materials

Soapberry fruits were harvested from Jianning County, Fujian Province, China (27°06′N, 117°25′E) at eight developmental stages (S1–8), covering the entire fruit development process. Three biological replicates were taken at each stage, for a total of 24 samples. For sRNA sequencing, pericarp samples were collected from three trees. The details of these materials were described in our previous report (Zhao et al., 2019; Xu et al., 2021b; Xu et al., 2022).

### sRNA library construction and high-throughput sequencing

Total RNA was extracted from the 24 samples using the FinePure Plant RNA Kit (Genfine Biotech, Beijing, China) in accordance with the manufacturer’s protocol. RNA quality and quantity were examined using the Agilent 2100 Bioanalyzer (Agilent Technologies, Santa Clara, CA, USA) and Nanodrop™ 2000 spectrophotometer (Thermo Fisher Scientific, Fremont, CA, USA). Sequencing libraries were constructed using the NEBNext^®^ Multiplex Small RNA Library Prep Set for Illumina^®^ (New England Biolabs, Ipswich, MA, USA) in accordance with the manufacturer’s protocol, and single-end sequencing was then performed on the Illumina HiSeq X Ten System (Illumina, San Diego, CA, USA) at ORI-BIO Co., Ltd. (Beijing, China). The sRNA dataset was deposited in the National Center for Biotechnology Information Databank at the Sequence Read Archive (SRA) database under accession numbers PRJNA784546, and includes 24 accession items (SRR17065996-SRR17065973).

### Identification of miRNAs and prediction of their targets

**Figure 1:**
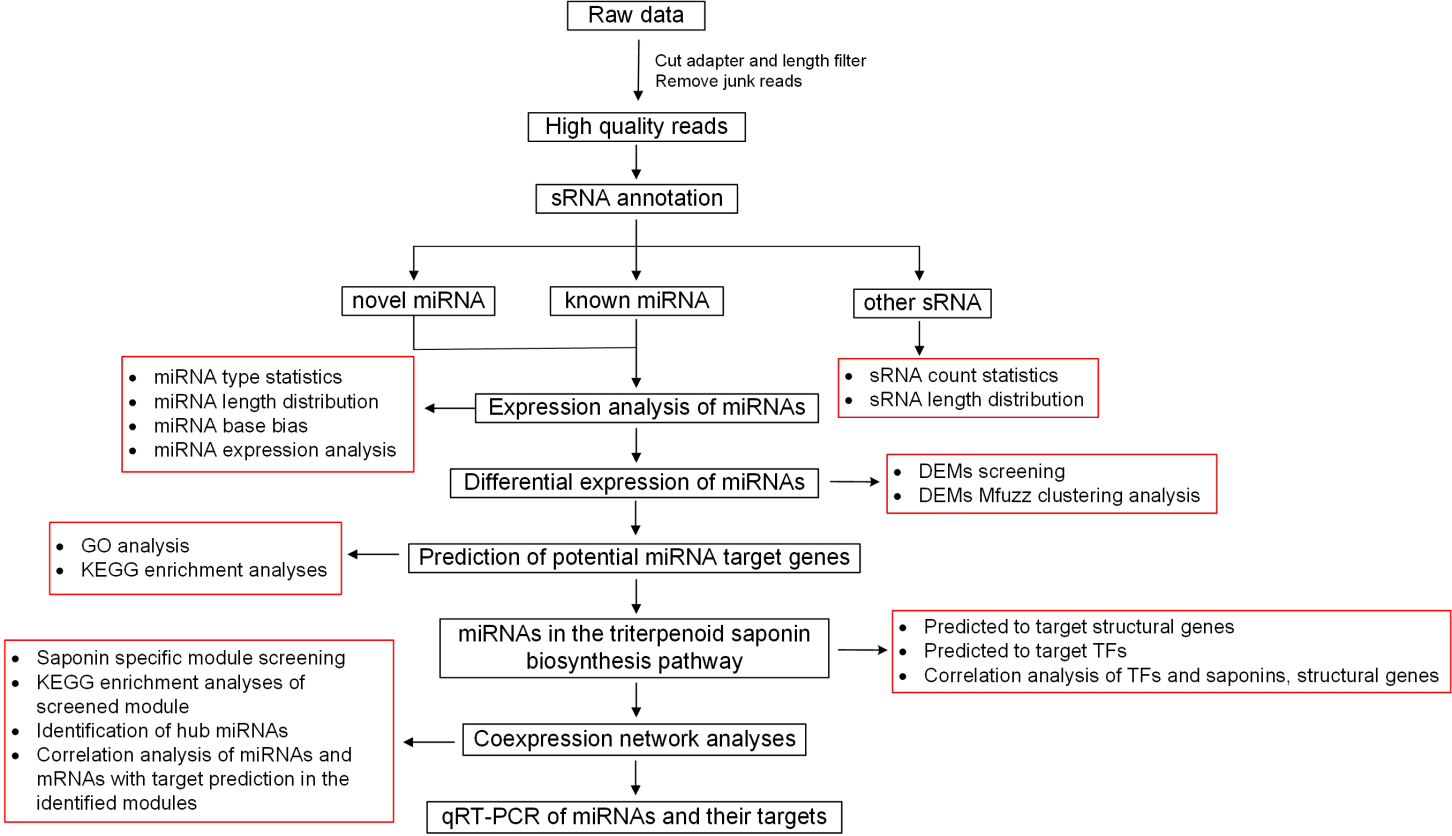
Methodology procedure chart.

The procedure for methodology is summarized in **Figure 1**. Briefly, after removal of adapter sequences and low-quality reads, the clean 15–34 nt sRNAs were mapped to the soapberry reference genome. The mapped sRNAs were then compared with sequences in the Rfam database (http://pfam.xfam.org/), allowing no more than two mismatches and 15 valid alignments to remove RNA categories including mRNA, tRNA, rRNA, snRNA, and snoRNA (Burge et al., 2013). The known miRNAs from mapped sRNAs were identified by BLASTN (http://blast.ncbi.nlm.nih.gov/Blast.cgi, Version 2.2.28+) search against the miRBase database (http://www.mirbase.org/) (Kozomara and Griffithsjones, 2014). To identify novel miRNAs, Bowtie2 (http://bowtie-bio.sourceforge.net/, Version 2.2.4) was used to map reads to the soapberry reference genome, and then the MIREAP program (http://mireap.sourceforge.net/, Version 0.2) was used to obtain all candidate precursors with hairpin-like structures (Langmead and Salzberg, 2012). Target genes of miRNAs, including known and novel miRNAs, were predicted using the MiRanda (http://www.microrna.org/microrna/, Version 3.3a; score=150, energy=-14) (Enright et al., 2003) and RNAhybrid (http://bibiserv.techfak.uni-bielefeld.de/rnahybrid/, Version 2.1.2; *E*-value < 1 × 10^−30^) (Rehmsmeier et al., 2004) programs from the soapberry transcriptome derived from the same eight developmental stages of soapberry pericarp, and the intersections of the predicted results were used as the final target gene prediction results. Target gene prediction was performed using the default parameters. In addition, annotation information for these target genes was extracted from the Soapberry Transcriptome Annotation Project (Xu et al., 2022). The Gene Ontology (GO) and Kyoto Encyclopedia of Genes and Genomes (KEGG) databases were used for gene annotation using emapper.py (EggNOG v5.0: http://eggnog5.embl.de/#/app/home) (Hernández-Plaza et al., 2022). In addition, GO and KEGG enrichment analyses were conducted using the clusterProfiler package in R.

### Differential expression analysis of miRNAs

Quantification of miRNA expression levels was based on reads per million (RPM): RPM= (mapped read counts/total read counts)×1000000. Differential expression analysis of miRNAs in samples from two pericarp development stages was performed using DESeq2 (Love et al., 2014). The *P*-values were calculated by displacement test and then corrected using the false discovery rate to calculate the *Q*-values. miRNAs with a *Q*-value < 0.05 and |log_2_(fold change)|>1 were identified as differentially expressed miRNAs (DEMs). Then, the online tool MISA-web was used to predict the simple sequence repeats (SSRs) in pre-mature sequences of identified DEMs using default parameters (Beier et al., 2017).

### miRNA coexpression network analysis

Coexpression networks were constructed using the WGCNA package in R (Langfelder and Horvath, 2008) and miRNA expression data from 24 libraries of soapberry. miRNAs with RPM values > 0 in more than two duplications were chosen for the WGCNA (Hollender et al., 2014). A gene expression adjacency matrix was generated to analyze the network topology with a soft threshold of β = 4, mergeCutHeight of 0.30, and minModuleSize of 30. The blockwiseModules were used with the default settings to obtain the modules. The first principal component module characteristic genes (MEs) were calculated to assess the expression levels of the gene modules. Then, module–trait associations were estimated by calculating the Pearson’s correlations between the MEs and the differentially accumulated saponin (DAS) content obtained from the pericarp materials from the same eight developmental stages determined previously by our group previously (**Table S1**). Integrative analysis was also performed to determine correlations between miRNA MEs and mRNA MEs from the same samples (Xu et al., 2022). Subsequently, we also performed Pearson’s correlation analysis to detect negatively associated miRNA–mRNA pairs. Cytoscape software (v. 3.8.2) was used to generate the networks (Shannon et al., 2003).

### qRT-PCR based expression analysis of miRNAs and their predicted targets

qRT-PCR was performed to validate the miRNAs identified using deep sequencing and to analyze their expression patterns. Total RNA was prepared as described above. For miRNA quantification, total RNA was polyadenylated and reverse transcribed into cDNA using *TransScript* ^®^ miRNA RT Enzyme Mix and 2×TS miRNA Reaction Mix (TransGen Biotech, Beijing, China). To quantify the expression of specific miRNA sequences, the cDNA samples were prepared with PerfectStart™ Green qPCR SuperMix (TransGen Biotech), ROX Reference Dye I (Thermo Fisher Scientific), Universal miRNA qPCR Primer (TransGen Biotech), and our miRNA-specific 5′ primer(s) and subjected to qPCR using the ABI StepOnePlus Real-Time PCR system (Applied Biosystems, Foster City, CA, USA). For target mRNA expression analysis, reverse transcription was performed using the Goldenstar RT6 cDNA Synthesis Kit ver. 2 (Tsingke, Beijing, China), followed by qPCR using TaKaRa TB Green™ Premix Ex Taq*™* (Tli RNaseH Plus; TaKaRa, Kyoto, Japan). Soapberry snRNA U6 (*Samuk01G0008400*) and *SmACT* (*Samuk13G0061200*) were used for normalization of the relative expression of miRNAs and mRNAs, respectively. The primers were designed using Oligo 7.0 software (**Table S2** and **S3**) and synthesized by Tsingke Biotech. Three biological and technical replicates were performed. The expression levels of miRNAs and their target genes were determined by calculating the fold change in selected stages of pericarp samples relative to stage S1 using the 2^−ΔΔCt^ method.

## Results

### High-throughput sequencing of sRNAs

**Figure 2:**
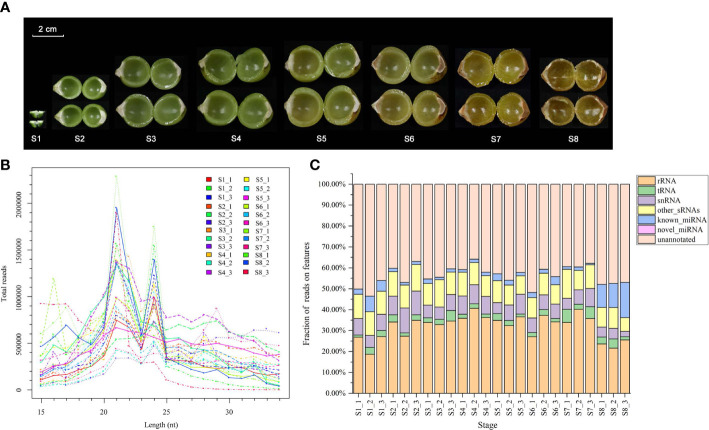
Diversity of sRNAs in soapberry. **(A)** Images of eight pericarp developmental stages from soapberry. **(B)** The length distribution of reads in the eight stages. **(C)** Small RNAs annotation in the Rfam databases. Fractions of different annotations are presented in a stacked bar plot.

Twenty-four sRNA libraries were constructed from soapberry pericarp samples and sequenced (**Figure 2A**). The raw reads yielded by deep sequencing of the 24 libraries ranged from 8.724 million (M) to 18.286 M (**Table S4**). After removing low-quality sequences and adaptor sequences, we obtained 5.576 M–14.134 M clean reads with average lengths of 19.2 nt–27.6 nt (**Table S4**). Q20 and Q30 base percentages of each library were > 99.0% (**Table S4**). The data obtained by sequencing were reliable and could be used for further experimental analyses.

The length distributions (15–34 nt) of all sRNA in the 24 samples revealed a major peak at 21 nt and a minor peak at 24 nt (**Figure 2B**). Unannotated sRNAs (35.85%–53.54%) and rRNAs (18.67%–40.65%) accounted for a considerable proportion of the total reads. A total of 57462–1359491 (0.72%–16.83%) sequences were produced as candidate miRNAs from the 24 libraries, including 57420–1358629 known miRNAs and 19–862 novel miRNAs (**Figure 2C**).

### Identification of miRNAs in soapberry

The size distribution of the predicted miRNAs differed among the 24 pericarp libraries, but the majority were between 15 and 34 nt in length, with 21 nt being the most common size followed by 20 nt (**Figure S1A**). Nucleotide bias analysis showed that these candidate miRNAs had a strong preference for adenosine (A) at position 15, for guanosine (G) at position 4 and 9, for cytidine (C) at position 19, and for uridine (U) at position 1 (**Figure S1B**). In total, 3036 miRNAs (2221 known miRNAs and 815 novel miRNAs) were predicted (**Table S5**; **Figure S2A**). Most of the identified miRNAs showed high degrees of identity among 60 plant species, including *Glycine max*, *Oryza sativa*, *Brachypodium distachyon*, *Medicago truncatula*, *Gossypium raimondii*, and *Arabidopsis lyrata* (**Figure S2B**). Particularly, 190 miRNAs identified in soapberry showed homology to those of *G. max*. Based on the sequence similarity, we grouped these known miRNAs into 1249 miRNA families. The largest families identified were miR156 with 59 members, followed by miR166 and miR169, with 44 and 41 members, respectively. Of the remaining 1246 families, 183 families were represented by 2–39 members, while 1063 were represented by only a single member (**Table S6**). The size of a miRNA family may represent its function.

The average expression level of known miRNAs (449.62 RPM/miRNA) was higher than that of novel miRNAs (1.70 RPM/miRNA), indicating that most novel miRNAs are weakly expressed and imprecisely processed (Cuperus et al., 2011). The expression levels of the known miRNAs markedly from < 5 to > 650000 RPM. In addition, 1266 known miRNA families were expressed during at least one pericarp development stage (**Table S7**), of which miR159 was the most abundant miRNA family. The expression levels of a few other miRNAs, including miR166, miR2916, miR482, miR396, miR162, miR894, and miR398, were also very high in both stages examined (RPM >2000). In addition, 56.99% of known miRNA families showed RPM values < 100. Meanwhile, some members of the same miRNA family also differed in expression level. For example, the RPM ranges of pta-miR159a, bdi-miR159b-3p.1, and lus-miR159c, belonging to the miR159 family, were 1.93–69.16, from 14.28–9215.03, and from 311.52–30461.96, respectively, in the eight development stages, indicating functional divergence of the different development stages of soapberry pericarp. In addition, the expression levels of novel miRNAs were low, and only novel-m1318-5p and novel-m0486-5p had RPM values > 100.

### Differential expression of miRNAs in soapberry

A total of 1372 DEMs were identified by pairwise comparisons, comprising 1171 known miRNAs (belong to 691 families) and 201 novel miRNAs. These DEMs belonged mainly to the miR156, miR166, miR159, miR396, and miR169 families. Compared with the previous stage, the number of downregulated miRNAs was more than that of upregulated miRNAs in each pairwise comparison (**Figure S3**). The 1372 DEMs were divided into 12 groups by Mfuzz clustering analysis based on their expression levels (**Figure S4**, **Table S8**). Among them, most miRNAs were preferentially expressed at a certain stage, e.g., cluster 2 (147 miRNAs) at stage S4 and cluster 3 (161 miRNAs) at stage S6. In addition, the expression of miRNAs in other clusters varied significantly among the different stages, e.g., cluster 1 (61 miRNAs) at stages S6 and S7 and cluster 6 (67 miRNAs) at stages S2 and S4 (**Figure S4**). Among the 1372 DEMs, only 12 miRNAs were predicted to contain SSR loci in their pre-mature sequences (**Table S9**).

### Prediction and annotation of potential miRNA target genes

**Figure 3:**
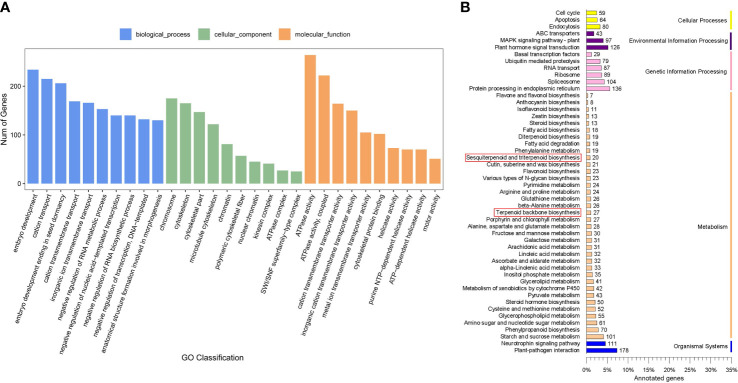
Target genes analysis of miRNAs. **(A)** GO enrichment analysis of target genes. **(B)** KEGG pathways analysis of target genes.

A total of 12750 genes were predicted to be the targets of 2188 miRNAs among the 3036 identified miRNAs, with an average of 5.83 targets per miRNA. These target genes were further subjected to GO analysis and KEGG pathway analyses. The target genes were classified into 2438, 392, and 653 GO terms for biological process, cellular component, and molecular function ontology, respectively. The main biological processes of miRNA target genes were cation transport, cation transmembrane transport, and negative regulation of RNA metabolic process. The molecular functions associated with the predicted miRNA targets were ATPase activity and cation transmembrane transporter activity, and chromosome was the main cellular component of miRNA target genes (**Figure 3A**). In addition, GO terms related to UDP-glycosyltransferase activity, UDP-glucosyltransferase activity, and transcription factor binding were also associated with the target genes. These observations suggested that the miRNA target genes related to transmembrane transport, UDP-glycosyltransferase activity, and transcription factor binding may play important roles in triterpenoid saponin biosynthesis. KEGG pathway enrichment analysis suggested that 47 mRNAs of miRNA target genes may function in terpenoid backbone biosynthesis and sesquiterpenoid and triterpenoid biosynthesis (**Figure 3B**). Among them, 46 mRNAs belonged to the target genes of DEMs such as ath-miR5021, han-miR3630-3p, and ppe-miR858, etc. (**Figure S5**). Furthermore, a variety of pathways associated with saponin transcription and transport, such as ABC transporters, plant hormone signal transduction, and basal transcription factors, were also found in this study (**Figure S5**), suggesting that some of the DEMs may play important roles in saponin biosynthesis in soapberry.

### Involvement of miRNAs in the triterpenoid saponin biosynthesis pathway

**Figure 4:**
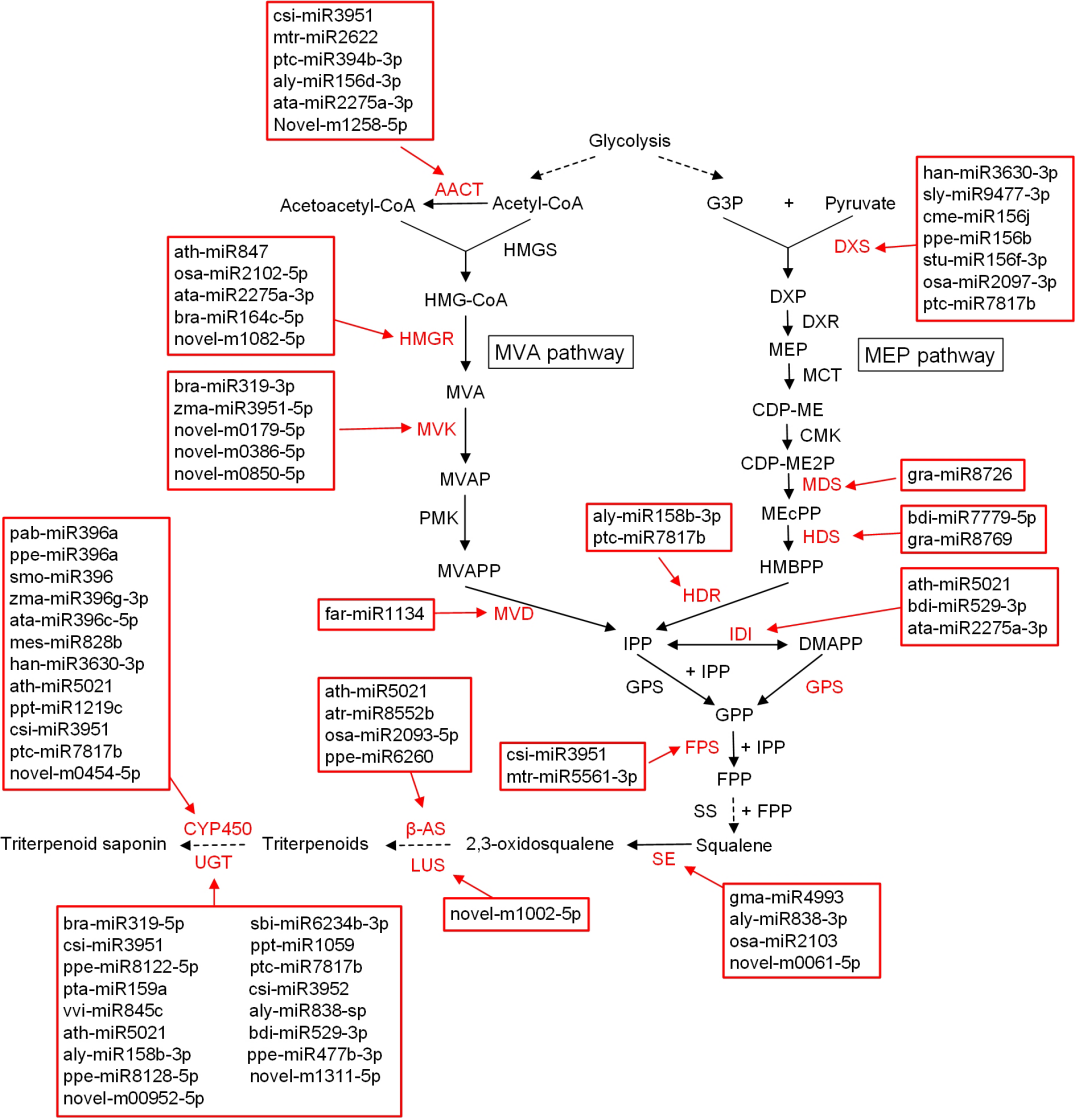
Hypothetical model of miRNAs regulation in triterpenoid biosynthesis pathway. AACT, acetyl-CoA C-acetyltransferase; HMGS, 3-hydroxyl-3-methylglutaryl-CoA synthase; HMG-CoA, 3-hydroxy-3-methylglutaryl-CoA; HMGR, 3-hydroxyl-3-methylglutaryl-CoA reductase; MVA, mevalonate; MVK, mevalolate kinase; MVAP, 5-metholvalic acid phosphoric; PMK, phosphoric acid valate kinase; MVAPP, 5-metholvalic acid pyrophosphoric acid; MVD, 5-hydroxyvalerate decarboxylase; G3P, 3-glyceraldehyde phosphate; DXS, 1-deoxy-D-xylulose-5-phosphate synthetase; DXP, 1-deoxy-D-xylulose-5-phosphate; DXR, 1-deoxy-D-xylulose-5-phosphate reductoisomerase; MEP, 2-C-methyl-D-erythritol-4-phosphATE; MCT, 4-cytidine diphosphate-2-C-methyl-D-erythritol synthetase; CDP-ME, 4-cytidine diphosphate-2-C-methyl-D-erythritol; CMK, 4-cytidine diphosphate-2-C-methyl-D-erythritol kinase; CDP-ME2P, 4-cytidine diphosphate-2-C-methyl-D-erythritol-2-phosphate; MDS, 2-C-methyl-D-erythritol-2,4-cyclic phosphate synthetase; MEcPP, 2-C-methyl-D-erythritol-2,4-cyclic phosphate; HDS, 4-hydroxy-3-methylbut-2-enyl diphosphate synthase; HMBPP, 4-hydroxy-3-methylbut-2-enyl pyrophosphate; HDR, 4-hydroxy-3-methylbut-2-enyl diphosphate reductases; IDI, isopentenyl pyrophosphate isomerase; IPP, isopentenyl pyrophosphate; DMAPP, dimethylallyl pyrophosphate; GPP, geranyl pyrophosphate; GPS, geranylpyrophosphate synthetase; FPS, farnesyl pyrophosphate synthetase; FPP, farnesyl pyrophosphate; SS, squalene epoxidase; β-AS, beta-amyrin synthase; LUS, lupeol synthase; CYP450, cytochrome P450; UGT, uridine diphosphate-dependent glycosyltransferases.

In the present study, 53 structural genes from 13 gene families involved in the saponin biosynthesis pathway were screened from miRNA target genes (**Figure S6**, **Table S10**). These candidate genes were predicted as targets of 58 miRNAs, totaling 91 miRNA–mRNA pairs (**Table S11**). Among them, 32 and 10 miRNAs were found to regulate terpenoid backbone biosynthesis and sesquiterpenoid and triterpenoid biosynthesis pathways, respectively (**Figure 4**). In addition, 26 miRNAs were predicted to target CYP450s and UGTs. Our results suggested that csi-miR3951, ath-miR5021, and ptc-miR7817b, each of which targets more than five saponin biosynthesis candidate genes in the miRNA–mRNA network (**Figure S6**), may be important regulators.

Furthermore, many TFs from bHLHs, bZIPs, ERFs, MYBs, and WRKYs, which are considered to play important roles in saponin biosynthesis, were also identified among the miRNA target genes (**Figure S7**, **Table S10**). In this study, 659 miRNA–TF pairs were obtained, including 314 miRNAs and 315 target TFs (including 43 bHLHs, 103 WRKYs, 26 bZIPs, 39 ERFs, and 104 MYBs) (**Table S11**). In addition, ppe-miR858, ptc-miR7817b, ath-miR5021, and csi-miR3951 were suggested to be important regulators, each of which targeted more than 20 TFs in the miRNA–mRNA network (**Figure S7**).

According to the FPKM and DAS content in the eight developmental stages of pericarp of soapberry, the correlation between the expression level of the selected TFs and structural genes or DAS content was analyzed. The 18 TFs which included 1 bHLH, 4 bZIPs, 4 ERFs, 4 MYBs, and 5 WRKYs (r > 0.8 or r < 0.8, P < 0.05; **Table S12**) were significantly correlated with DAS content. In addition, 63 TFs (11 bHLHs, 4 bZIPs, 7 ERFs, 22 MYBs, and 19 WRKYs) associated with the regulation of 30 structural genes (r > 0.8 or r < 0.8, P < 0.05; **Table S12**). After taking out the redundancy, 75 TFs that were associated with the biosynthesis of saponin.

### Coexpression network analyses

**Figure 5:**
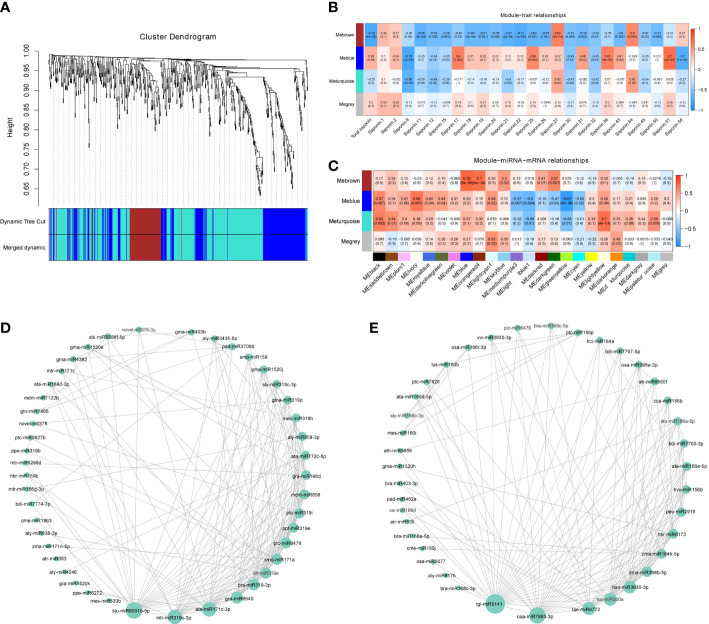
WGCNA of expressed miRNAs. **(A)** Hierarchical clustering tree (cluster dendrogram) showing four modules of coexpressed miRNAs by WGCNA. Each leaf of tree corresponds to one miRNA. The major tree branches constitute four modules, labeled with different colors. **(B)** Module–saponin relationship. Each row represents a module. Each column represents a specific saponin. The value in each cell at the row-column intersection represents the correlation coefficient between the module and the saponin and is displayed according to the color scale on the right. The value in parentheses in each cell represents the *P*-value. **(C)** Correlation and *P*-values of miRNA modules eigengenes with mRNAs modules eigengenes from the same pericarp samples in soapberry. The rows represent miRNA modules and the columns represent mRNA modules. The correlation networks of miRNAs in the Mebrown **(D)** and Meblue **(E)** modules, in which only edges with weight above a threshold of 0.10 and 0.25 are displayed, respectively.

Next, we used the miRNA gene expression profile data as the original data for WGCNA. After elimination of deletions and outlier values, 616 original miRNAs remained. These miRNAs were clustered into four tree branches, and each of which represented a module (labeled with different colors, with the grey module containing the uncorrelated genes) (**Figure 5A** and **Table S13**). The number of miRNAs (module size) ranged from 80 (Mebrown) to 325 (Meturquoise) miRNAs for each module, with an average size of 205 miRNAs (**Table S13**). Module–trait relationship analysis showed that the Mebrown module with 80 miRNAs was highly negatively correlated with total saponin and 15 other saponins, including the major saponins: saponin 11, saponin 19, saponin 20, and saponin 22 (**Figure 5B**, **Table S1**). The Meblue module contained 204 miRNAs and showed a significant association with saponin 9, saponin 17, saponin 25, saponin 39, saponin 51, and saponin 54 (**Figure 5B**). In addition, as miRNAs can negatively regulate mRNA abundance, miRNA MEs were correlated with mRNA MEs from the same pericarp samples in soapberry. According to the results of our previous mRNA module–saponin correlation analysis, the MEblue and MEgreenyellow modules were significantly correlated with most saponins. As shown in **Figure 5C**, the mRNA module (MEgreenyellow) was highly negatively correlated to one miRNA module (Meblue). Thus, two miRNA modules (Mebrown and Meblue) were finally extracted for further study.

### KEGG enrichment analysis of target genes of miRNAs in the identified modules

There were 1432 and 3128 predicted target genes in the mRNA transcriptomes of the same pericarp samples in the miRNA Mebrown and Meblue modules, respectively. The results of KEGG enrichment analysis showed that transcription machinery and glycosyltransferases were significantly enriched in the Mebrown module (**Figure S8A**). In addition, KEGG pathways related to sesquiterpenoid and triterpenoid biosynthesis, terpenoid backbone biosynthesis, and cytochrome P450 were also involved. In the Meblue module, the target genes were significantly associated with messenger RNA biogenesis, transcription machinery, and ABC transporters (**Figure S8B**). In addition, nine saponin biosynthetic structural genes, including 3-hydroxy-3-methylglutaryl-CoA reductase (*HMGR*), mevalonate kinase (*MVK*), 1-deoxy-D-xylulose-5-phosphate synthetase (*DXS*), 2-C-methyl-D-erythritol-2,4-cyclic phosphate synthetase (*MDS*), isopentenyl pyrophosphate isomerase (*IDI*), *FPS*, and *β-AS*, were also associated with terpenoid backbone biosynthesis and sesquiterpenoid and triterpenoid biosynthesis (**Figure S8B**). Another six saponin biosynthesis candidate genes, including CYP450s and UGTs, were also found among the target genes of miRNAs in the Meblue module.

### Identification of hub miRNAs

In the Mebrown module, 45 miRNAs showed high correlations (edge weight ≥ 0.10), and stu-miR8001b-5p, mtr-miR319c-3p, ata-miR171c-3p, gra-miR8640, and bra-miR319-3p were identified as the top five hub miRNAs (**Figure 5D**). A total of 59 genes, including 1 structural gene and 7 TFs, were predicted to be targets of these hub miRNAs (**Table S14**). Among them, bra-miR319-3p was predicted to target a mevalonate kinase (*SmMVK2*) in the mevalonic acid pathway and a TFs of the WRKY family (*SmWRKY101*).

The network diagram of the Meblue module was composed of 39 coexpressed miRNAs (edge weight ≥ 0.25), of which rgl-miR5141, osa-miR7693-3p, tae-miR9773, han-miR3630-3p, and tcc-miR530a had the highest degrees of connectivity (**Figure 5E**). In total, 257 genes, including 4 structural genes and 17 TFs, were predicted to be targets of these five miRNAs (**Table S14**). Of these, han-miR3630-3p was predicted to target one *DXS*, three *CYP450s*, and one *bHLH*, seven *WRKYs*, and two *MYBs*. In addition, tcc-miR530a targeted *SmbHLH29*, a bHLH family TF.

Most of the hub miRNAs in the Mebrown and Meblue modules identified in the WGCNA were also identified as DEMs among the various developmental stages. These results suggested that WGCNA and differential expression analysis identified not only similar biological processes associated with saponin biosynthesis but also the same miRNAs.

### Integration of negative correlations between miRNAs and mRNAs with target prediction in the identified modules

**Figure 6:**
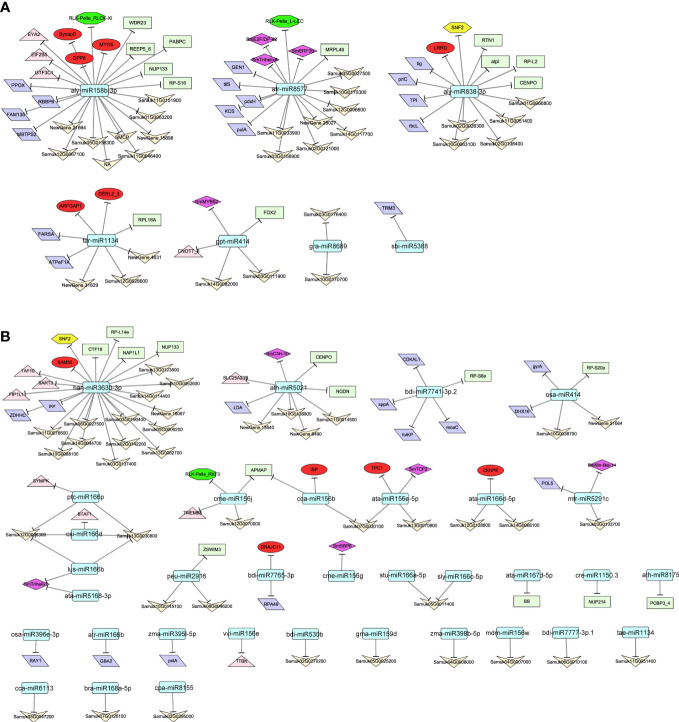
Regulatory network of negatively–correlated miRNAs and mRNAs in identified modules. miRNAs in modules Mebrown **(A)** and Meblue **(B)** that were significantly associated with saponin biosynthesis and were negatively correlated with various mRNAs as indicated by the arrows. Round rectangle, diamond, hexagon, octagon, ellipse, parallelogram, rectangle, triangle, and V represent miRNA, TFs, TRs, PKs, transporters, enzymes, proteins, others and unknow genes, respectively.

The correlations between mRNA and miRNA expression levels were analyzed according to the transcriptomic data from the eight developmental stages of soapberry pericarp (r < −0.4, *P* < 0.05). In total, 74 and 94 significant negative miRNA−target gene pairs were identified in modules Mebrown and Meblue, respectively (**Table S15**). In module Mebrown, a total of seven conserved miRNAs were predicted to target 74 genes (**Figure 6A**). In addition, aly-miR158b-3p had the largest number of target genes, and followed by atr-miR8577, aly-miR838-3p, and far-miR1134. Two TFs that may be related to saponins biosynthesis, *SmERF39* and *SmMYB52*, were the targets of atr-miR8577 and ppt-miR414, respectively. In addition, 86 genes were negatively correlated with 34 miRNAs in the Meblue module (**Figure 6B**). Of these, the TFs from the TCP, Trihelix, C3H, SBP, and Nin-like families, were targets of ata-miR156e-5p, ata-miR5168-3p, ath-miR5021, cme-miR156g, lus-miR166b, and mtr-miR5291c. In addition to TFs, miRNA has also been found to regulate other regulators (e.g., transcriptional regulators (TRs), protein kinases (PKs), transporters), enzymes, proteins, and other genes with unknown functions in the identified modules. These genes may also play important roles in the biosynthesis of triterpenoid saponin in soapberry, and further studies are therefore required.

### qRT-PCR analysis of the miRNAs and their targets

**Figure 7:**
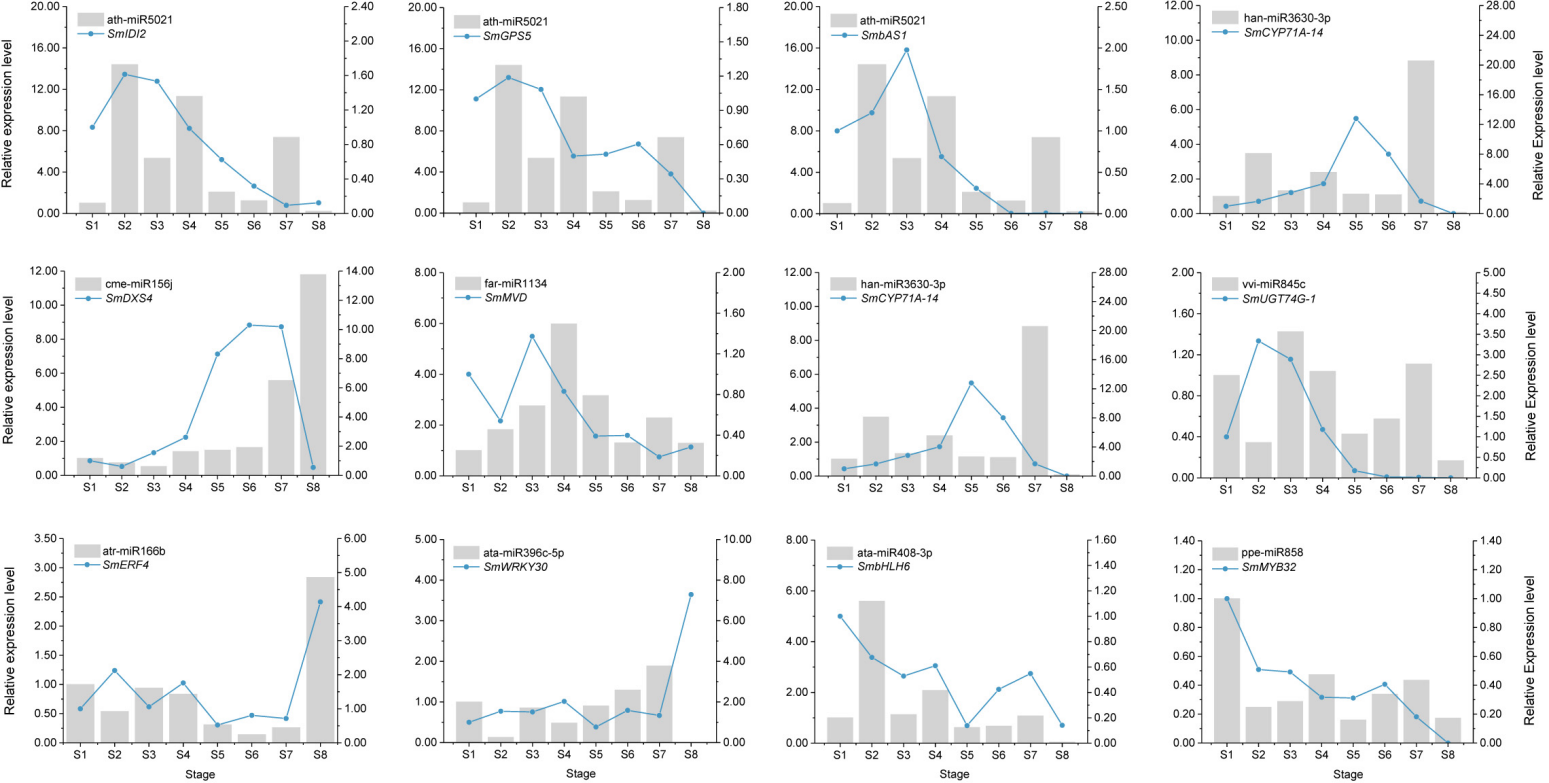
Expression correlation of miRNAs and their targets. The bars and lines indicate the relative expression level of miRNAs and their corresponding targets in the eight developmental stages of soapberry pericarps. The Y-axis on the left and right represents the abundance of the miRNAs and their targets, respectively. SnRNA U6 and *SmACT* were used for normalizing the relative expression of miRNAs and their targets, respectively. The expression level of the miRNAs and their corresponding targets in the stage S1 were set as 1.0. Relative expression level was calculated using the 2^–ΔΔ^ *^C^* ^t^ method. Data indicate the mean values of three biological replications.

From the high-throughput sequencing results, we randomly selected nine miRNAs and their corresponding 12 target genes (8 structural genes and 4 TFs) for qRT-PCR. The results indicated that the relative expression levels of these miRNAs at eight developmental stages were essentially consistent with the sRNA-Seq data (**Figure S9A**). In addition, the expression patterns of 12 target genes also showed strong correlations between the qRT-PCR and RNA-Seq data (**Figure S9B**). These results further suggested that our high-throughput sequencing data were reliable for evaluating miRNA–mRNA regulatory network in soapberry. Furthermore, the differential expression trends of these nine miRNAs were measured by qRT-PCR. The expression levels of two miRNAs, ath-miR5021 and ata-miR408-3, peaked at stage S2, which two other miRNAs (atr-miR166b and cme-miR156j) showed the highest expression at stage S8. These results suggest that different miRNAs may regulate saponin biosynthesis at different stages. Simultaneously, the expression patterns of these miRNAs were negatively correlated with their corresponding target genes at most pericarp developmental stages (**Figure 7**). For example, the level of cme-miR156j expression was higher at stage S8, followed by stages S7 and S6. The corresponding target gene, *SmDXS4* showed the reverse trend. The expression levels of ata-miR396c-5p and *SmWRKY30* also showed negative correlations at most pericarp developmental stages. However, in the case of these 12 miRNA–mRNA pairs, the expression of the target gene was partially positively correlated with the expression of the miRNA at different stages (**Figure 7**). The relative changes in the expression of miRNAs and their target genes were not identical, indicating that each target gene can be regulated by many miRNAs. These relationships between miRNAs and their targets suggested that miRNAs play important roles in the regulation of genes involved in plant saponin biosynthesis.

## Discussion

### sRNA deep sequencing of soapberry pericarps at eight fruit growth stages

Triterpenoid saponin are important bioactive components of soapberry pericarp. Accumulating evidence suggests that miRNAs play important regulatory roles in the biosynthesis of secondary metabolites. For example, miR1134, miR5021 and miR7539 affect the accumulation of terpenoids in *O. sativa* by targeting upstream genes (*DXS*, *HMGR*, *IDS*, and *IDI*) in the terpenoid biosynthesis pathway (Gupta et al., 2017). Yu et al. (2015) reported that miR156 directly binds and activates the promoter of the terpene synthase 21 gene, which mediates sesquiterpene biosynthesis in *Arabidopsis* and *Pogostemon cablin*. However, little is known about the miRNAs related to the regulation of triterpenoid biosynthesis in soapberry. In this study, we identified some miRNAs and their target genes associated with saponin biosynthesis, thus providing valuable information regarding the molecular regulatory mechanism of triterpenoid saponin biosynthesis in soapberry. This study generated more than 226.67 M clean reads. Of these, a major peak occurred at 21 nt with a minor peak at 24 nt, consistent with the distribution patterns of sRNAs reported in other plants, such as *Ginkgo biloba* (Ye et al., 2020), *Camellia sinensis* (Zheng et al., 2015) and *Citrus junos* (Xie et al., 2017). In this study, a total of 3036 miRNAs (2221 known miRNAs and 815 novel miRNAs) were identified. The reads of known miRNAs ranged from a few to hundreds of thousands, similar to previous studies in *Solanum lycopersicum* and *O. sativa* (Cheah et al., 2015; Candar-Cakir et al., 2016). In addition, 56.99% of known miRNA families exhibited low abundance (RPM < 100) in soapberry pericarp, and only a few miRNA families were highly expressed, as in the reports of other plant species, including *C. sinensis* (Guo et al., 2017), *Arachis hypogaea* (Zhao et al., 2010), and *Gossypium hirsutum* (Xie et al., 2015). The miRNA expression patterns are highly conserved in different plants, with most members of the miR156, miR159, miR166, and miR398 families being highly expressed in most plants, including *O. sativa*, *Triticum aestivum*, and *P. notoginseng* (Devi et al., 2013; Wang et al., 2014; Wei et al., 2015). We found that miR159, miR166, miR2916, miR482, miR396, miR162, miR894, and miR398 were the eight most abundantly expressed miRNA families in soapberry pericarp, similar to the results reported in other plants. Some miRNA families are monocot- or dicot- specific (Mandhan et al., 2012), as further demonstrated by the presence of miR403 (dicot-specific miRNA) in soapberry. In the present study, with pericarp development, the RPM value of miR156 increased from 175 (stage S1) to 35499 (stage S8), while the RPM value of miR172 decreased from 92 (stage S1) to 5 (stage S8) (**Table S7**), consistent with the previous results showing the opposite regulation of these two miRNAs (Mandhan et al., 2012).

### Presence of many novel miRNAs in soapberry

Although early research showed that most plant miRNAs have been conserved throughout evolution, increasing numbers of novel miRNAs with tissue-specific, stage-specific, and low expression have been revealed by sRNA sequencing (Zhao et al., 2012). A total of 815 novel miRNAs were predicted in soapberry by bioinformatics analyses in the present study, and most of these miRNAs begin with 5′-uridine, which is consistent with typical miRNA sequences (Ye et al., 2020). As expected, most of the novel miRNAs had a low expression, in agreement with the previous results in *Arabidopsis* and *S. lycopersicum* (Fahlgren et al., 2007; Cao et al., 2014). Therefore, there are still many unannotated sequences in these 24 libraries, of which 35.85%–53.54% of the reads were unannotated. We speculate that many low-abundance non-coding RNAs remain to be discovered.

### Identified differential expression of miRNA

A variety of miRNAs are involved in the biosynthesis of plant secondary metabolites. In addition, some miRNAs show significant increases or decreases in expression in major organs or at major developmental stages (Hao et al., 2012; Chen et al., 2020). We identified 1372 DEMs at different pericarp developmental stages. Several stage-specific miRNAs were also detected in this study. We divided these 1372 DEMs into 12 clusters by Mfuzz clustering analysis, and most were preferentially expressed at one or two stages, suggesting that stage-specific expression of the DEMs reflects their specific biological functions (**Figure S4**). The expression of known miRNAs, such as miR156, miR854, miR172, and miR166, changed significantly during the pericarp development, and these miRNAs were predicted to target genes involved in regulation of the saponin biosynthesis pathway. These miRNAs were also identified in *P. notoginseng*, *P. ginseng*, and *C. borivilianum* (Mathiyalagan et al., 2013; Wei et al., 2015; Kajal and Singh, 2017).

### miRNA targets structural genes and TFs related to saponin biosynthesis in soapberry

Identification of the target genes of the identified miRNAs will help elucidate their roles in various metabolic pathways (Samad et al., 2019). Many conserved miRNAs have the same or homologous targets in different plants, indicating their conserved function in specific biological processes (Liu et al., 2017). Singh et al. (2016) predicted that miR156 targets *DXS*, which is involved in the 2-C-methyl-D-erythritol 4-phosphate pathway and governs terpenoid biosynthesis in *Mentha* spp. We also reached a similar conclusion in soapberry. In our study, three miRNAs (cme-miR156j, ppe-miR156b, and stu-miR156f-3p) were predicted to target *SmDXS4*. qRT-PCR analyses further showed that cme-miR156j negatively regulated the expression of *SmDXS4* at most pericarp developmental stages (**Figure 7**). In addition, regulation of terpenoid biosynthesis by miR5021 has been observed in previous studies. For example, the regulation of *HMGR* and *IDI* by miR5021 was reported in *Xanthium strumarium* (Fan et al., 2015), and miR5021 in *Catharanthus roseus* homologous to *Arabidopsis* was predicted to target *HDS*, which is involved in terpenoid biosynthesis (Pani and Mahapatra, 2013). In the present study, ath-miR5021 was predicted to target *SmIDI1*, *SmIDI2*, *SmGPS5*, *SmbAS1*, *SmCYP71D-3*, and *SmUGT74G-2* in soapberry. Further, we also verified that ath-miR5021 was inversely correlated with *SmIDI2*, *SmGPS5*, *SmbAS1*, and *SmCYP71D-3* expression by qRT-PCR at most developmental stages (**Figure 7**). Taken together with the results of previous studies, we inferred that cme-miR156j and ath-miR5021 are involved in the saponin biosynthesis in soapberry pericarp.

TFs are proteins that bind to specific DNA sequences, usually motifs within the promoters of target genes, to control their transcription (Ye et al., 2020). Several studies have suggested that miRNAs can target TFs that control plant secondary metabolite biosynthesis, including anthocyanin (Gou et al., 2011) and sesquiterpenoid (Yu et al., 2015). In most cases, highly conserved miRNAs regulate homologous targets encoding TFs at the same target sites in every plant species in which they exist (Gao et al., 2018). In the present study, at least five types of TFs (bHLH, WRKY, bZIP, ERF, and MYB) were predicted to be miRNA targets associated with saponin biosynthesis, involving 158 miRNA families (miR858, miR482, miR7817, miR5021, miR166, miR3951, and miR159, etc.). Among these TFs, several bHLHs have been shown to play crucial roles in the biosynthesis of triterpenoid saponin. For example, two bHLH TFs, TSAR1 and TSAR2, were shown to participate in the biosynthesis of nonhemolytic soyasaponins and hemolytic soyasaponins in *M. truncatula*, respectively (Mertens et al., 2016). GubHLH3 was shown to activate the transcription of the soyasaponin biosynthetic genes *CYP93E3* and *CYP72A566* in *Glycyrrhiza uralensis*, and overexpression of *GubHLH3* in transgenic *G. uralensis* hairy roots enhanced the levels of *β-AS*, *CYP93E3*, and *CYP72A566* transcripts (Tamura et al., 2018). Here, 54 bHLH genes were found to be targeted by 43 known miRNAs (including miR319, miR159, miR156, and miR408) and 11 novel miRNAs, indicating their roles in triterpenoid saponin biosynthesis in soapberry. This was similar to previous observations that miR156 and miR159 play important roles in regulating secondary metabolite biosynthesis by targeting bHLH TFs (Gou et al., 2011; Song et al., 2011). Furthermore, other TFs important for saponin biosynthesis, such as WRKY, bZIP, ERF, and MYB, were also shown to be miRNA targets (Xu et al., 2021a). Our study suggested that MYBs may be the targets of miR159, miR166, miR482, miR319, and miR858 in soapberry. Previous studies have also indicated that this is also the case in *Mentha spicata* (Reddy et al., 2017), *Arabidopsis thaliana* (Sharma et al., 2016), and *Malus domestica* (apple) (Xia et al., 2012). We also predicted that atr-miR166b targets SmERF4, ata-miR396c-5p targets SmWRKY30, ata-miR408-3p targets SmbHLH6, and ppe-miR858 targets SmMYB32. The results of qRT-PCR analyses confirmed that atr-miR166b, ata-miR396c-5p, ata-miR408-3p, and ppe-miR858 negatively regulate the expression of SmERF4, SmWRKY30, SmbHLH6, and SmMYB32, respectively (**Figure 7**). Taken together, these observations suggest that the predicted miRNAs are likely to target a variety of mRNAs encoding TFs. miRNAs have established a complex and diverse network to regulate saponin biosynthesis in soapberry pericarp, but further studies are needed to fully clarify the specific mechanisms of regulating this response.

### Regulatory networks participated in saponin biosynthesis

A great deal of evidence suggests that a group of miRNAs (clusters and families) may be involved in the regulation of a group of common targets (Ponsuksili et al., 2013) and are related to phenotypes. Here, WGCNA was performed to cluster miRNA products and revealed two modules (Mebrown and Meblue) significantly correlated with saponins. The Meblue module was also significantly negatively correlated with a mRNA module (MEgreenyellow). KEGG enrichment analysis showed that target genes from the Mebrown and Meblue modules were associated with transcription machinery and ABC transporters, respectively. Meanwhile, the pathways of sesquiterpenoid and triterpenoid biosynthesis and terpenoid backbone biosynthesis were also associated with the target genes. The miRNAs in these two selected modules may play important regulatory roles in saponin biosynthesis and transport. Furthermore, hub miRNAs are highly interconnected nodes in the network and may play regulatory roles in the common coexpressed network (Nunez et al., 2013). In our study, two hub miRNAs in MEbrown, mtr-miR319c-3p and bra-miR319-3p, were predicted to target *SmWRKY101* and *SmMVK2*, respectively. A hub miRNA in MEblue, han-miR3630-3p, was predicted to target a *DXS*, three *CYP450s*, one *bHLH*, seven *WRKYs*, and two *MYBs*. In addition, these three hub miRNAs were also identified as the DEMs during pericarp development. The results of qRT-PCR analyses showed an inverse correlation between han-miR3630-3p and *SmCYP71A-14* levels at most pericarp developmental stages. Based on the described functions of these miRNAs in this study, these hub miRNAs may be important for saponin biosynthesis in soapberry.

In addition, we found many miRNAs that were negatively correlated with their target genes in the modules Mebrown and Meblue. In the Mebrown module, genes that may participate in saponin biosynthesis, such as TFs, TRs, PKs, and transporters, were significantly negatively correlated with, and some of them were predicted to be targets of, atr-miR8577, aly-miR838-3p, aly-miR158b-3p, and far-miR1134. Many genes of the Meblue module were predicted to be associated with saponin biosynthesis, including six TFs, one TR, one PK, and five transporters. These genes were significantly negatively correlated with—and were predicted to be targets of—miRNAs, such as ath-miR5021, han-miR3630-3p, and cme-miR156j. Similarly, other miRNAs, including gra-miR8689, osa-miR414, and ptc-miR166p, were significantly negatively correlated with many enzymes, proteins, and other genes of unknown function, which may also play important roles in the biosynthesis of triterpenoid saponin in soapberry. However, although some miRNAs in the network have biological functions described in the literature, some do not have documented functions. It is possible that these unknown miRNAs may participate in the same pathways together with known miRNAs to regulate specific functions or pathways related to saponin biosynthesis.

Although the typical effect of miRNAs is to downregulate mRNA expression, there are also cases of positive miRNA regulation (Guo et al., 2010). In the present study, some miRNAs were not negatively correlated with their target genes, and qRT-PCR also showed a lack of negative correlations of the relative expression of eight miRNA with that of their target gene pairs at certain stages. However, the positive correlations may also reflect secondary miRNA targets (Nunez et al., 2013) or adaptive target miRNA responses (Mamdani et al., 2015). Therefore, some interacting miRNA–mRNA pairs may not be negatively correlated, and this requires specific analysis based on specific situations.

## Conclusions

This is the first study to investigate the miRNA expression profiles of soapberry pericarp. We identified 3036 miRNAs, including 2221 known miRNAs and 815 novel miRNAs, in soapberry pericarp, as well as their target genes. KEGG enrichment analysis suggested that some of the differentially expressed miRNAs, including ath-miR5021_R4-18L20, han-miR3630-3p_R5-20L22, and ppe-miR858_R3-18L21, may be involved in terpenoid backbone biosynthesis and sesquiterpenoid and triterpenoid biosynthesis pathways in soapberry pericarp. We constructed two coexpression miRNA modules/networks that were highly correlated with variations in the saponin contents or highly negatively correlated with the mRNA modules. Within the two networks, we identified 10 key candidate miRNAs that were weighted as module hub miRNAs. Furthermore, multiple miRNA–mRNA regulatory models were proposed, especially ath-miR5021–*SmIDI2*/*SmGPS5*/*SmbAS1*/*SmCYP71D-3*/*SmUGT74G-2*, cme-miR156j–*SmDXS4*/*SmbHLH18*, han-miR3630-3p–*SmCYP71A-14*/*SmbHLH54*/*SmMYB135*/*SmWRKY32*, atr-miR8577–*SmERF39*, ppe-miR858–*SmMYB5*/*SmMYB32*, and ppt-miR414–*SmMYB52*. Some of these miRNAs and their targets were validated by qRT-PCR. This study provided new evidence for miRNA regulation in triterpenoid saponin biosynthesis in soapberry and identified potential targets for designing experiments to regulate saponin content.

## Data availability statement

The datasets presented in this study can be found in online repositories. The names of the repository/repositories and accession number(s) can be found in the article/**Supplementary Material**.

## Author contributions

YX, LJ and ZC conceived the research and designed the experiments. YX and JL wrote the manuscript. XJ, XW and LW analyzed the date. YH, YHG, SG and XHW performed the experiments. GZ, TZ and YG modified the language and revised the manuscript. All authors contributed to the article and approved the submitted version.
