# Supplementary material for: Integrative analysis of microRNAs and mRNAs reveals the regulatory networks of triterpenoid saponin metabolism in Soapberry (Sapindus mukorossi Gaertn.)
Source: Front Plant Sci. 2023 Jan 9;13:1037784. doi: 10.3389/fpls.2022.1037784 (PMC9869041; doi:10.3389/fpls.2022.1037784)
Supplement: Supplementary file 1 [file DataSheet_1.docx]

# Supporting Information

Integrative analysis of microRNAs and mRNAs reveals the regulatory networks of triterpenoid saponin metabolism in Soapberry (*Sapindus mukorossi* Gaertn.)

Yuanyuan Xu^1,2,3†^, Jiming Liu^1,2,3†^, Xiangqin Ji^4^, Guochun Zhao^1,2,3^, Tianyun Zhao^1,2,3^, Xin Wang^1,2,3^, Lixian Wang^1,2,3^, Shilun Gao^1,2,3^, Yingying Hao^1,2,3^, Yuhan Gao^1,2,3^, Yuan Gao^5^, Xuehuang Weng^6^, Liming Jia^1,2,3*^, Zhong Chen^1,2,3,7*^

^1^ Key Laboratory of Silviculture and Conservation of the Ministry of Education, College of Forestry, Beijing Forestry University, Beijing 100083, China

^2^ National Energy R&D Center for Non-food Biomass, Beijing Forestry University, Beijing 100083, China

^3^ National Innovation Alliance of Sapindus Industry, Beijing Forestry University, Beijing 100083, China

^4^ Hangzhou KaiTai Biotechnology Co., Ltd, Hangzhou, Zhejiang 310030, China

^5^ Planning and Design Institute of Forest Products Industry, National Forestry and Grassland Administration, Beijing 100010, China

^6^ Yuanhua Forestry Biological Technology Co., Ltd., Sanming, Fujian 354500, China

^7^ Beijing Advanced Innovation Center for Tree Breeding by Molecular Design, Beijing Forestry University, Beijing 100083, China

†These authors contributed equally: Yuanyuan Xu, Jiming Liu.

*Corresponding authors:

Liming Jia: [jlm@bjfu.edu.cn](mailto:jlm@bjfu.edu.cn);

Zhong Chen: zhongchen@bjfu.edu.cn.

College of Forestry, Beijing Forestry University, 35 E Qinghua Road, Beijing 100083, China

Tel.: +86-13501103773 (L. Jia); +86-13020086124 (Z. Chen)

**Supplementary Figures**


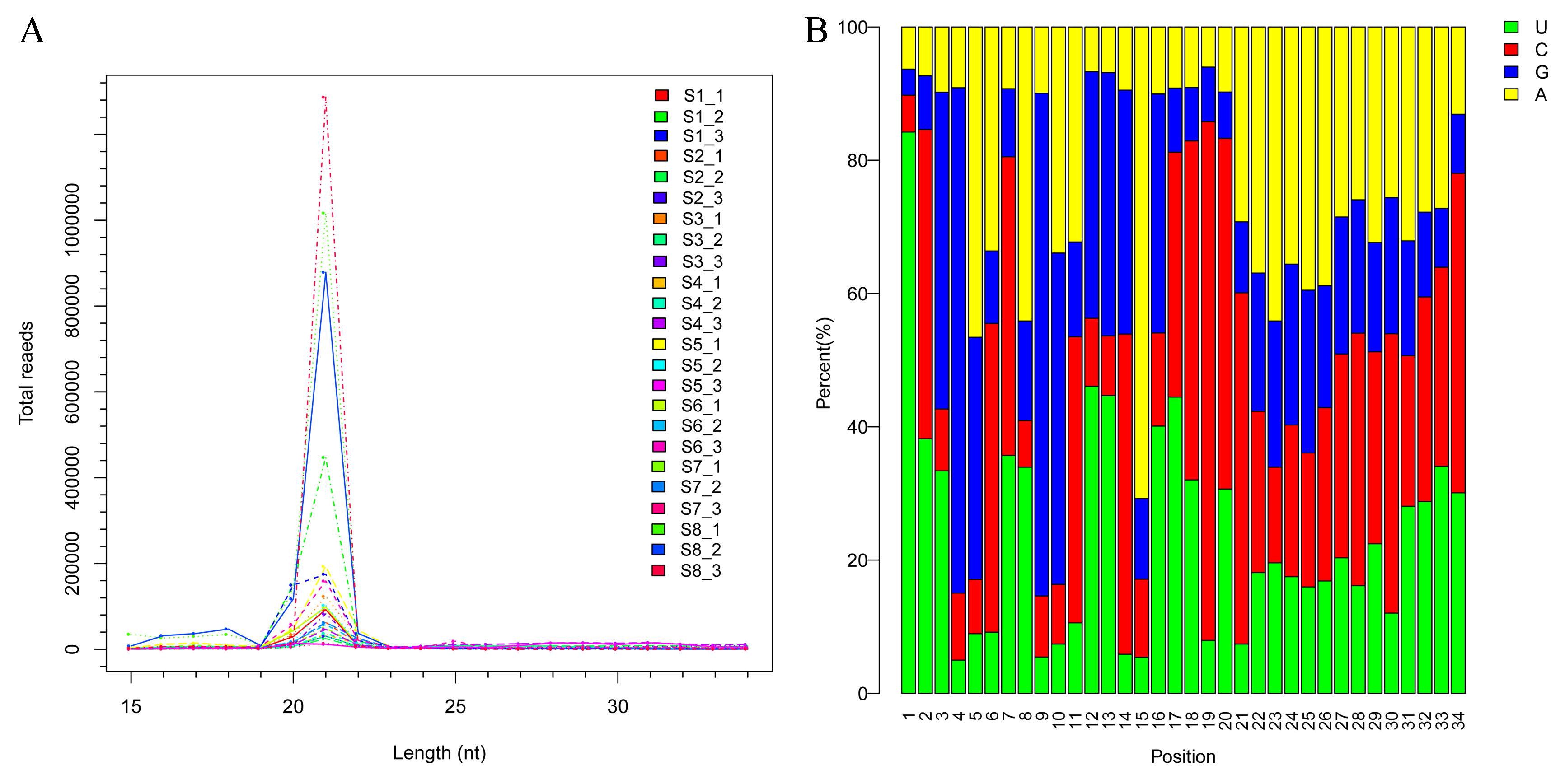


**Fig. S1 Length distribution of miRNA and bias of miRNA bases.** (A) Length distribution of prediction miRNA from soapberry. (B) Distribution of the miRNA bases at each position.


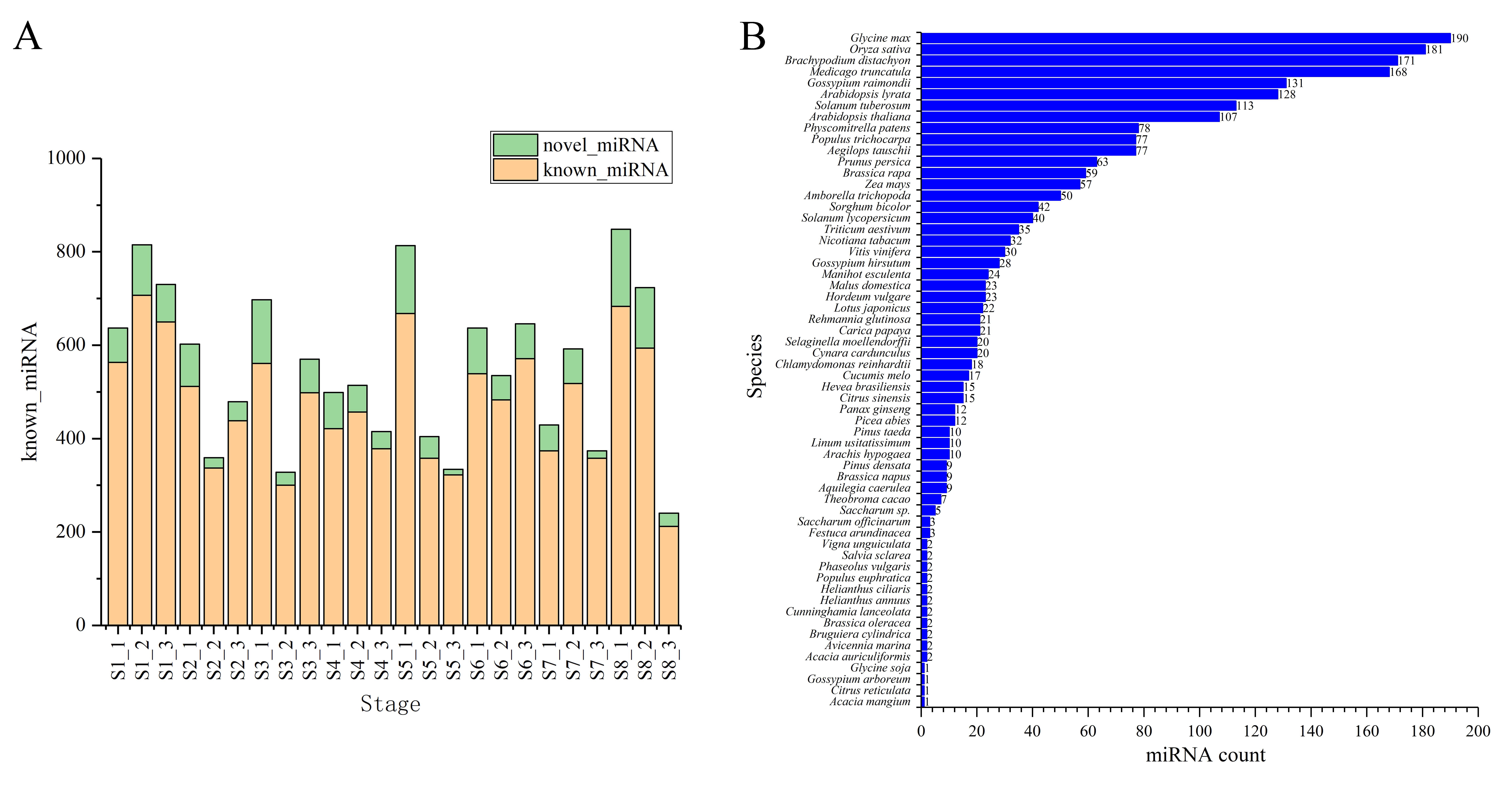


**Fig. S2 Identification of miRNAs.** (A) Number of known and novel miRNAs in different libraries. (B) Conservation of the identified known miRNAs in soapberry.


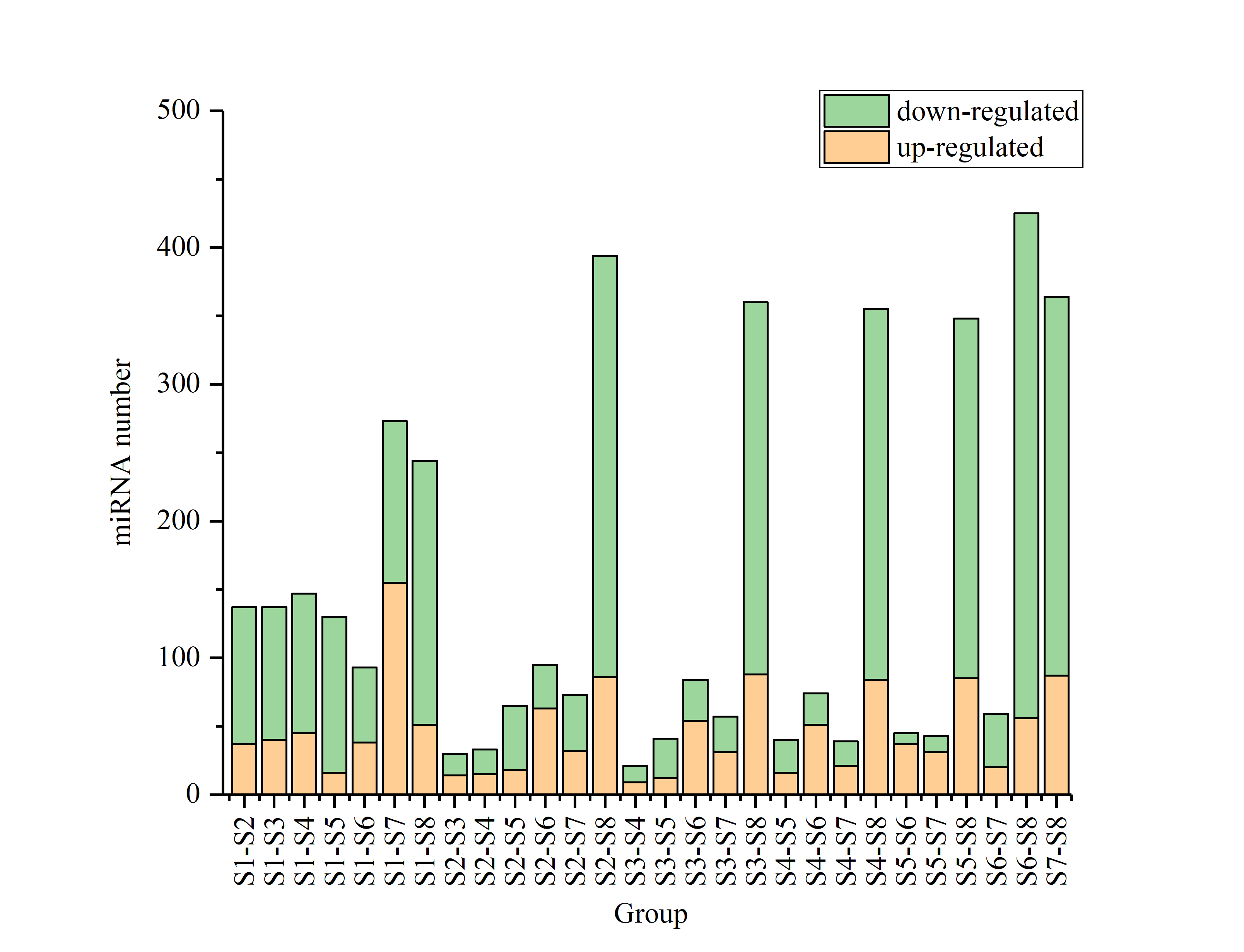


**Fig. S3 Number of DEMs between two tested groups**


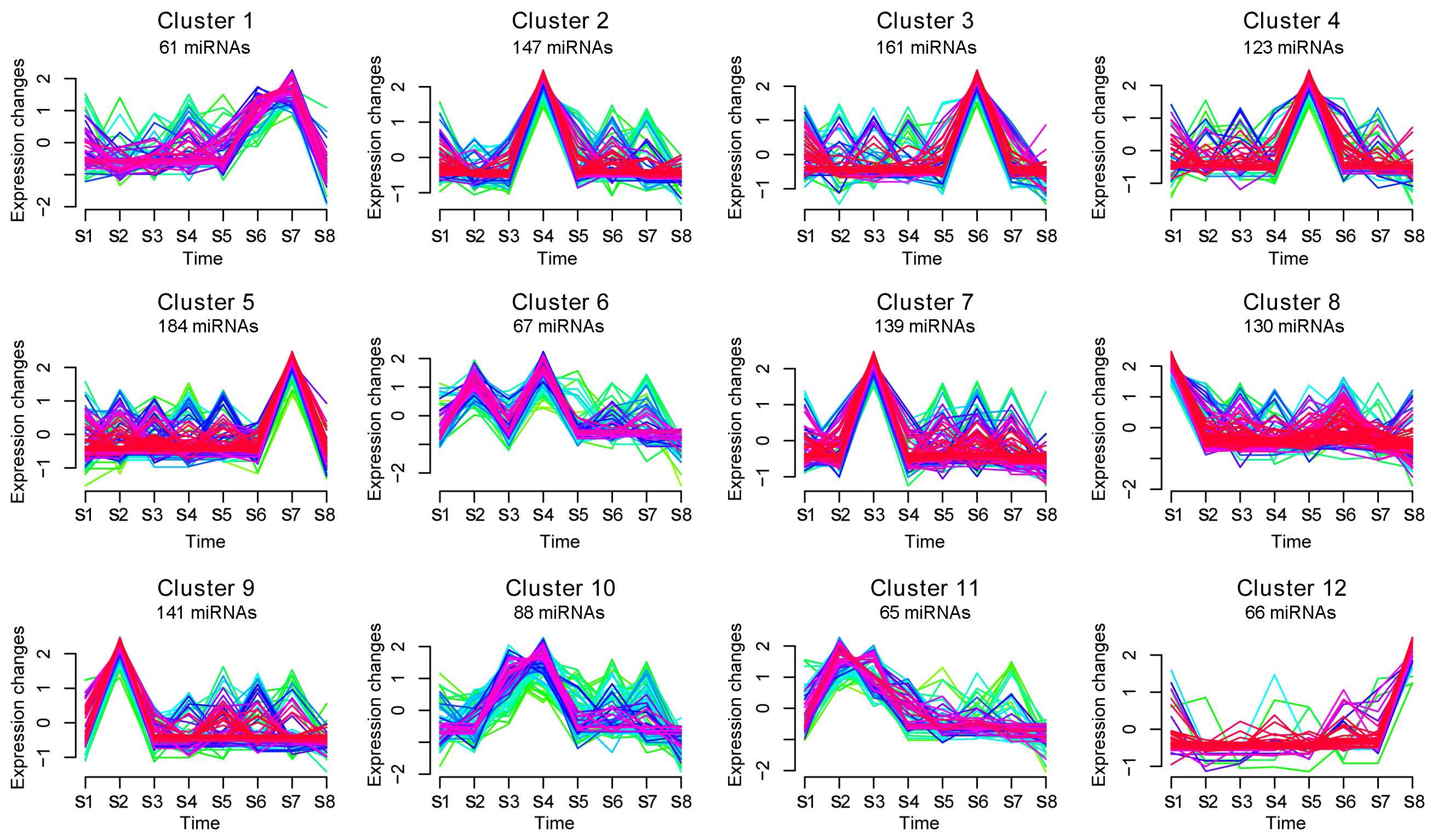


**Fig. S4 Results of the Mfuzz clustering of 1372 DEMs on their expression patterns**


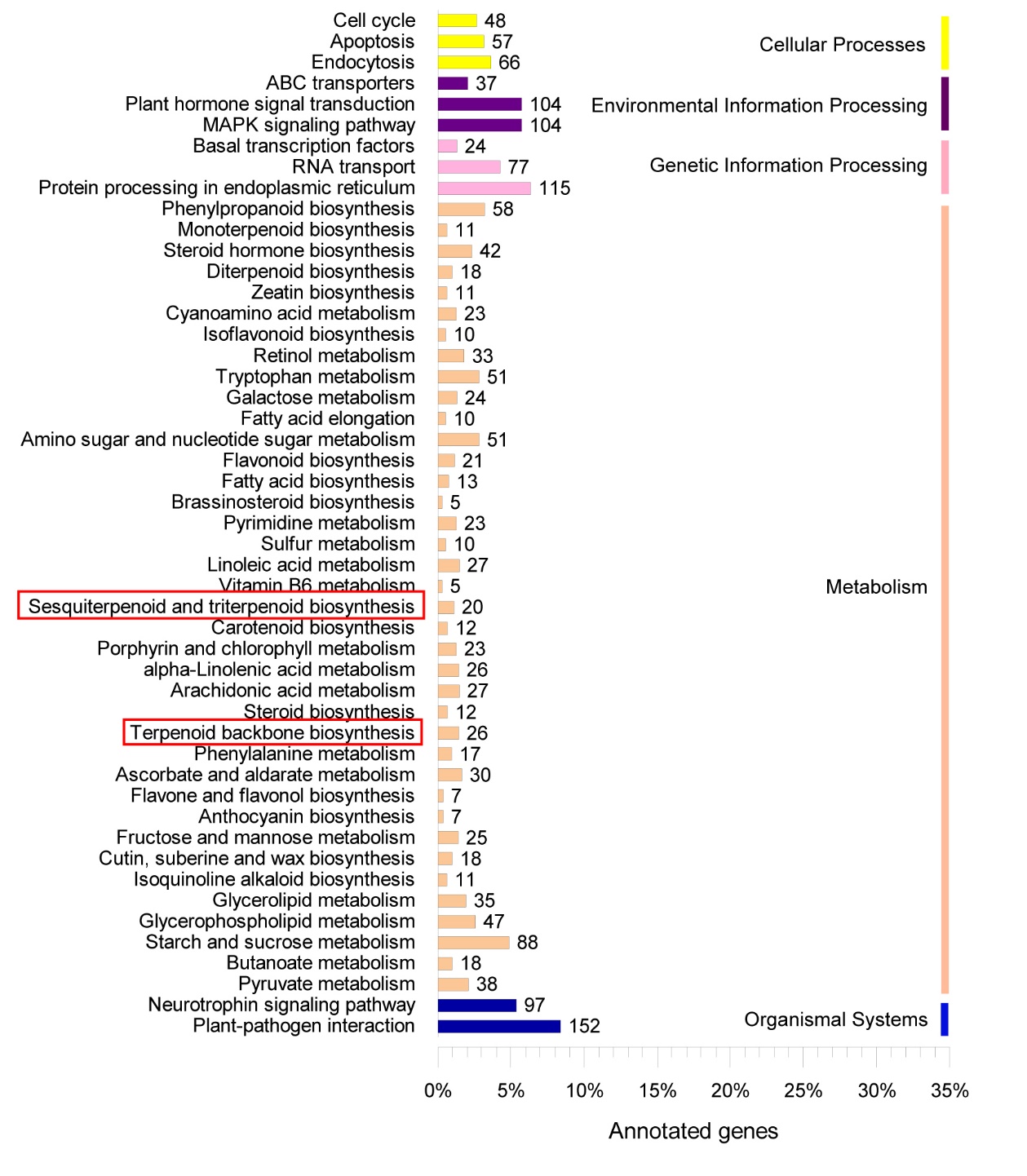


**Fig. S5 KEGG pathways analysis of DEM target genes**


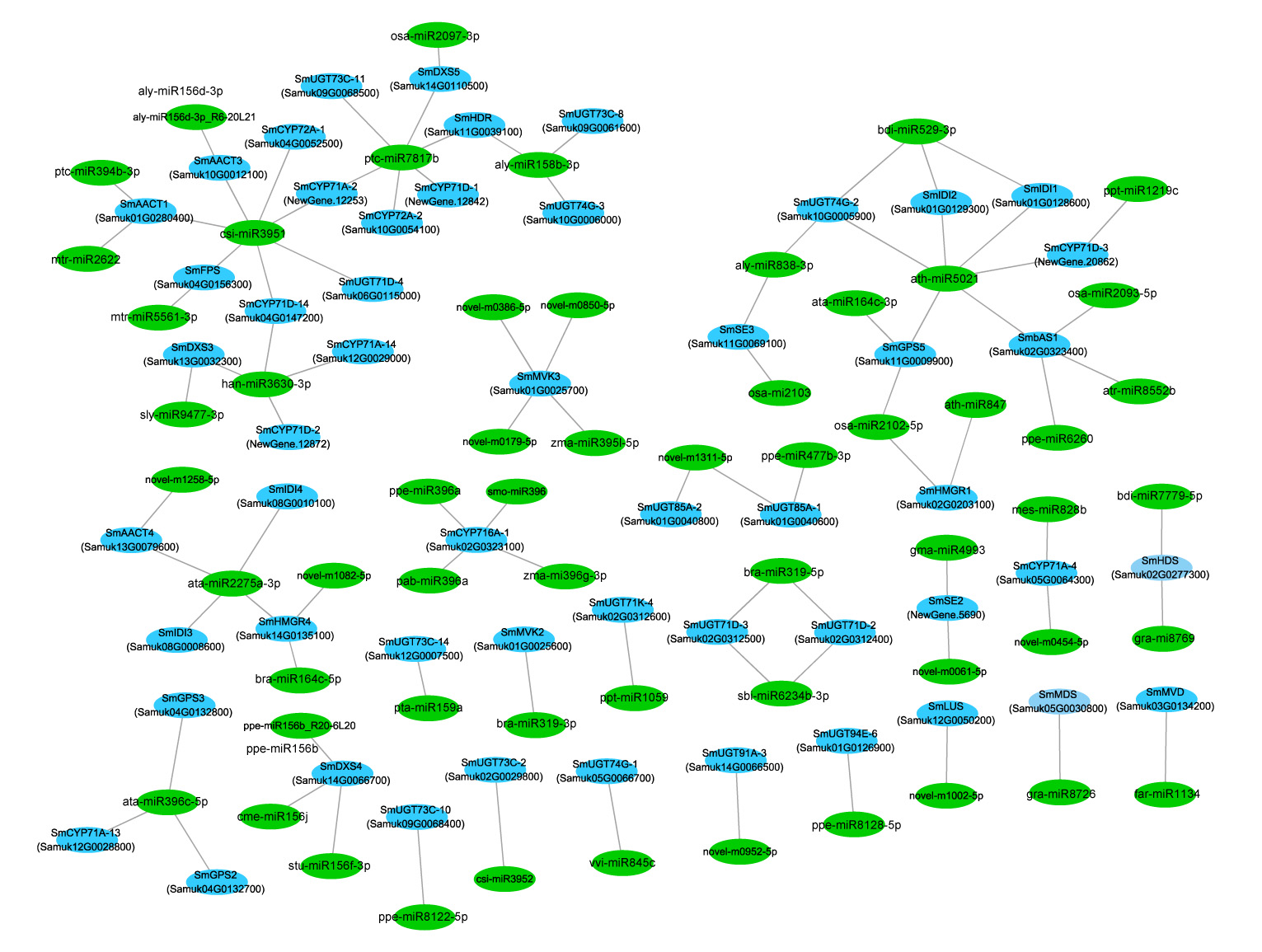


**Fig. S6 The miRNA-mRNA regulatory network of candidate genes in the saponins biosynthesis of soapberry**


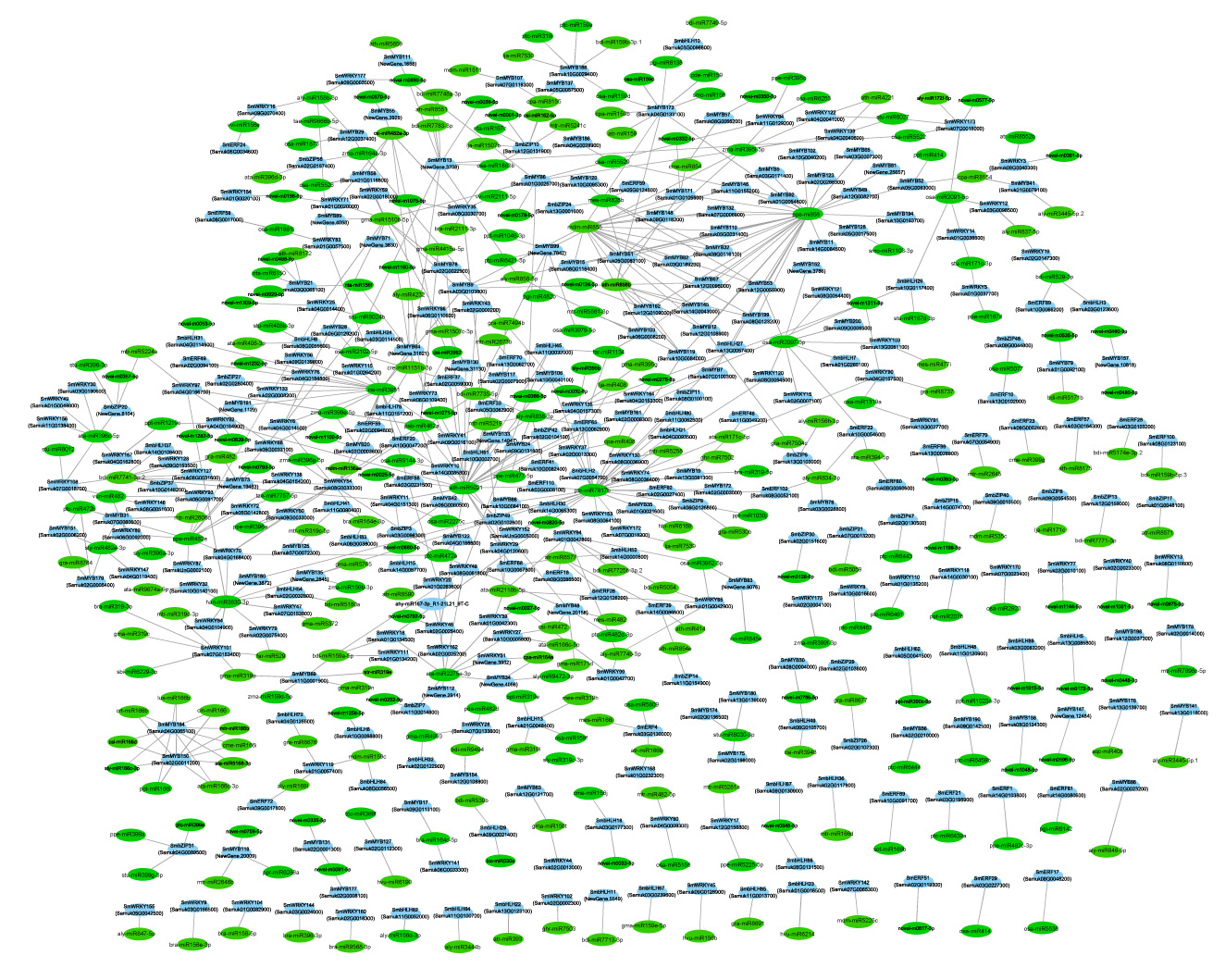


**Fig. S7 The miRNA-mRNA regulatory network of transcription factor may be involved in the saponins biosynthesis of soapberry**


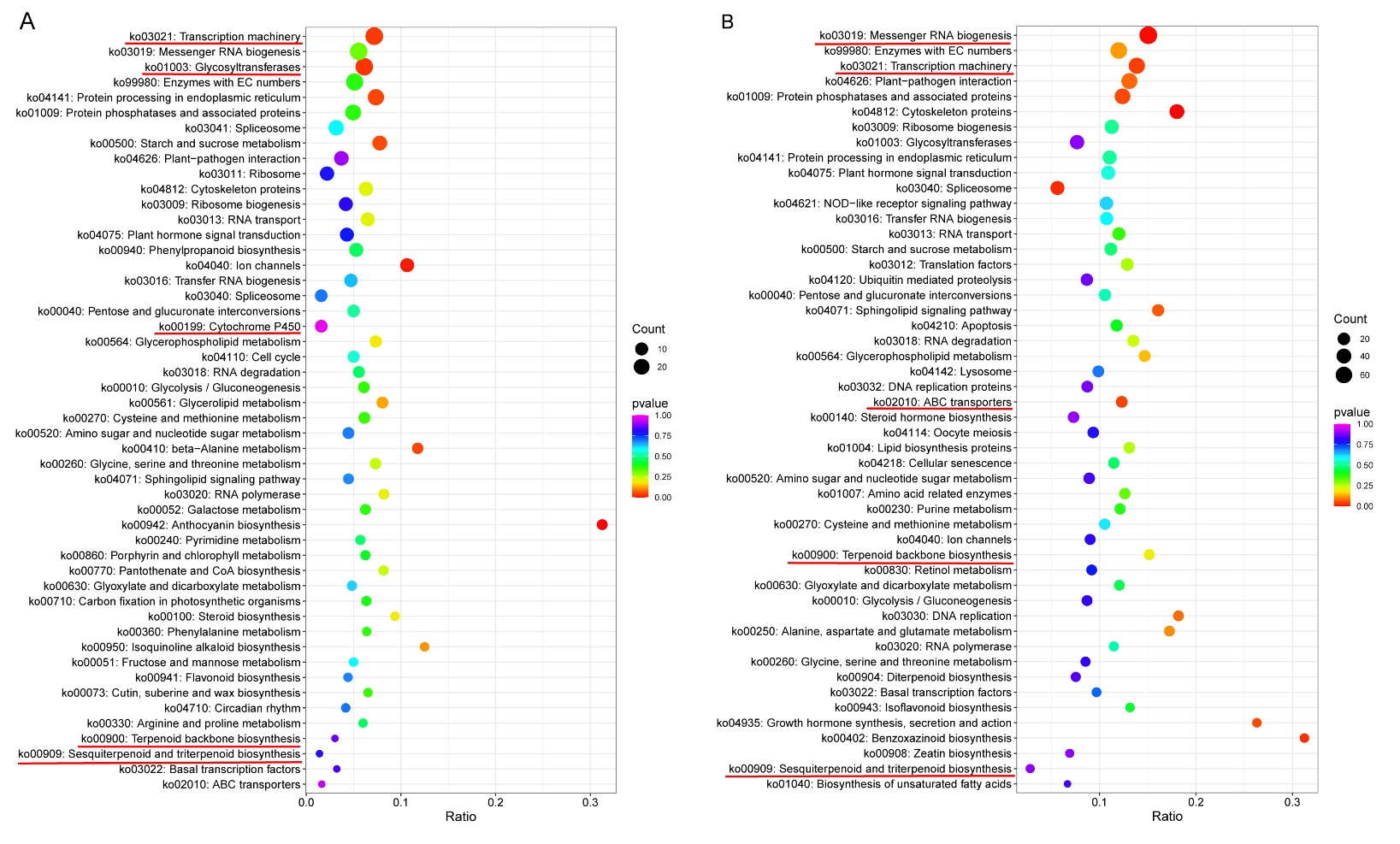


**Fig. S8 KEGG enrichment analysis of the target genes of the two associated miRNA modules.** The KEGG enrichment analysis of the target genes in the Mebrown (A) and Meblue (B) modules showed the top 50 pathways with the most significant enrichment.


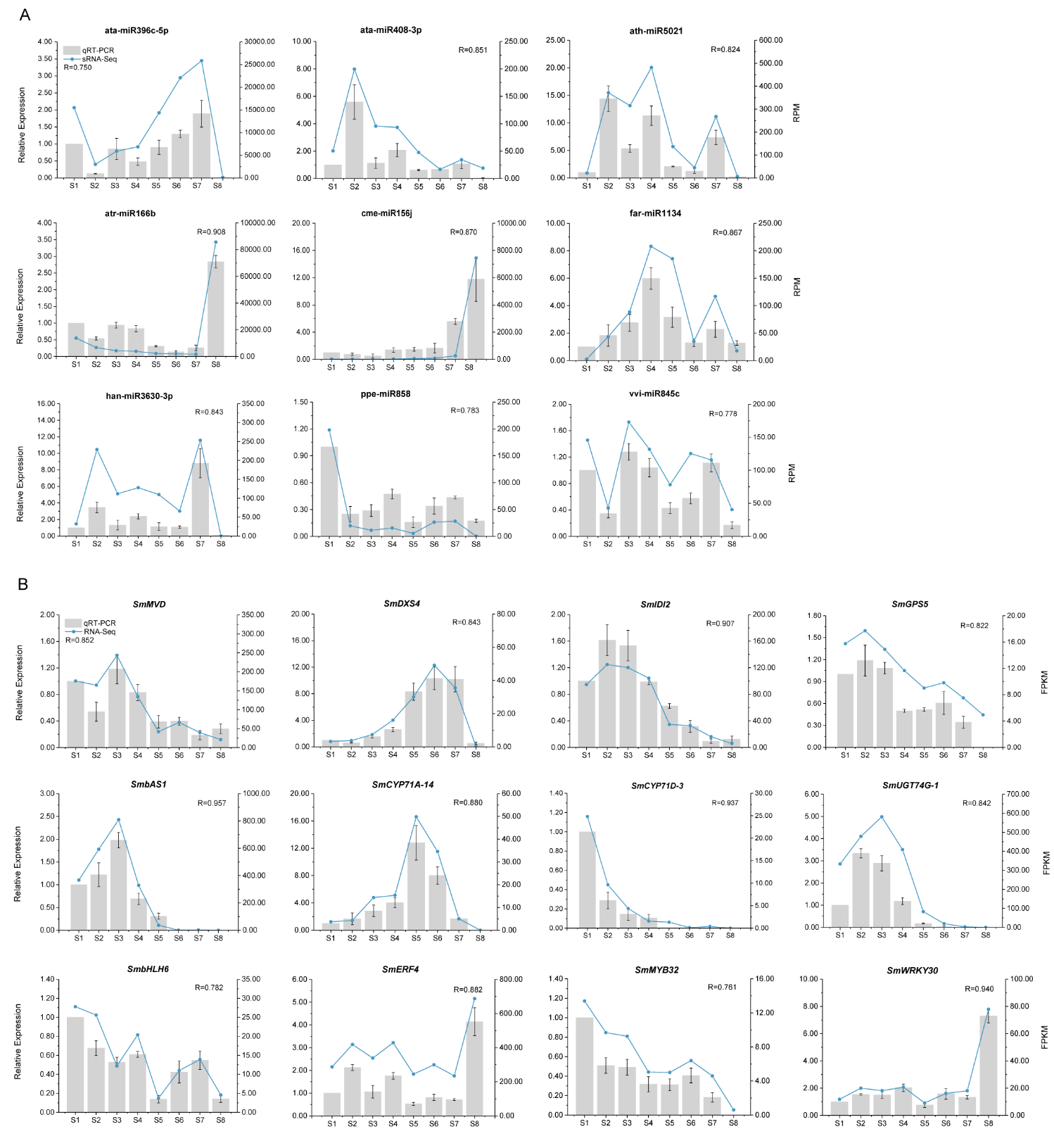


**Fig. S9 Validation of nine miRNAs and 12 corresponding targets (eight structural genes and four TFs) by qRT-PCR.** (A) Relative expression levels of eight miRNAs. Bars and Lines indicate relative expression level of qRT-PCR and RPM of sRNA-Seq, respectively. The Y-axis on the left and right represents the relative expression level of of qRT-PCR and RPM of sRNA-Seq, respectively. (B) Relative expression levels of eight targets. Bars and Lines indicate relative expression level of qRT-PCR and FPKM of RNA-Seq, respectively. The Y-axis on the left and right represents the relative expression level of of qRT-PCR and FPKM of RNA-Seq, respectively. SnRNA U6 and *SmACT* were used for normalizing the relative expression of miRNAs and their targets, respectively. The expression level of the miRNAs and their corresponding targets in the stage S1 were set as 1.0. Relative expression level was calculated using the 2–ΔΔ*C*t method. Data indicate the mean values of three biological replicates.
